# Retraction: Daphnetin inhibits proliferation and glycolysis in colorectal cancer cells by regulating the PI3K/Akt signaling pathway

**DOI:** 10.1039/d1ra90033a

**Published:** 2021-01-26

**Authors:** Laura Fisher

**Affiliations:** Royal Society of Chemistry Thomas Graham House, Science Park, Milton Road Cambridge CB4 0WF UK advances-rsc@rsc.org

## Abstract

Retraction of ‘Daphnetin inhibits proliferation and glycolysis in colorectal cancer cells by regulating the PI3K/Akt signaling pathway’ by Zhikuan He *et al.*, *RSC Adv.*, 2018, **8**, 34483–34490, DOI: 10.1039/C8RA05583A.

The Royal Society of Chemistry hereby wholly retracts this *RSC Advances* article due to concerns with the reliability of the data. The images in the article, and the raw data provided by the authors, were screened by an image integrity expert. The western blots in the figures are all over-contrasted and the shape of many of the individual bands is unusual with uncharacteristic blunt ends. In addition, while the original data provided by the authors matched the figures, it could not be counted as raw data as the images had been cut and were not un-processed. Therefore, the blots in the figures and the raw data do not appear genuine. Furthermore, the raw data provided by the authors was found to closely resemble raw data provided for a number of other articles, which is unexpected given that there are completely different author lists for these articles.

Given the significance of the concerns about the validity of both the data in the article and the raw data provided by the authors, the findings presented in this paper are not reliable.

The authors have been informed but have not responded to any correspondence regarding the retraction.

Signed: Laura Fisher, Executive Editor, *RSC Advances*

Date: 15^th^ January 2021

## Supplementary Material

